# Congenital Muscular Atony

**Published:** 1909-04-24

**Authors:** 


### Notes on Current Neurology.

### CONGENITAL MUSCULAR ATONY.

Congenital muscular atony was first described by Oppenheim in 1900. A recent number of the *Gazette des Hôpitaux* contains a summary from the pen of Dr. Levi Sirugue of the present state of our knowledge of this disease. It is characterised by the following symptoms. It appears at birth, though it may at that time escape notice, and would seem to be in no way a family disease. Muscular atony is present without paralysis, and the limbs, especially the lower limbs, are the parts usually attacked. Sometimes, however, the muscles of the head and neck are affected. There is an abnormal laxity of the muscles, which allows of great exaggeration of passive movements. The eye muscles, those used for suction and deglutition, the respiratory muscles and the sphincters are, as a rule, unaffected. The muscles affected lack tone, but show no atrophy. Sometimes a hard, sleek, white œdema is present, called by Kundt "fatty pseudo-hypertrophy," and affects most commonly the vulva, thighs, and buttocks. Tendinous and cutaneous reflexes are almost totally abolished, but electrical and mechanical excitability of the muscles persists, and there is no reaction of degeneration. There is an absence of any troubles of sensibility, mentality, and functions of the senses. The evolution of the disease is retrogressive, and would seem, in typical cases, the exact opposite of that of Little's disease. As a rule, the muscles of the extremities, and principally those of the upper extremity, are the first to resume a normal condition, and the flexors, being attacked to a less degree than the extensors, recover their strength the earlier. Although the prognosis is for the most part good, a few cases are recorded in which death ensued from broncho-pneumonia, hypostatic congestion, etc. There is still some obscurity as to the pathology of the disease. Oppenheim thought that it is due to a retardation in the development of the muscles, and that there is no disease of the central nervous system except a functional derangement of the anterior horn-cells. Bing, on the contrary, thinks that there is an arrested development of the medullo-cerebellar paths. Badonin is of opinion that the defect is in the peripheral neurone. Other observers have put it down to disease of the thymus, and noted the relation between a persistent thymus and the grave myasthenias. Again, the œdematous condition observed at times, leads Berti to think that it is akin to myxœdema. Yet another theory, that of Cattaneo, maintains that it is due to a lesion of some glands which exert an internal secretion. Treatment is usually effective, and causes rapid amelioration of the condition without, in the present state of our knowledge, leading to a complete cure. Since, however, the disease has only been seen in very young children, it must be cured spontaneously with age. Elctrical treatment is efficacious, especially the faradic current. Baths and massage with arsenic and strychnine are useful adjuvants. The danger, espeoially with very young children, lies in the vulnerability of the respiratory system.

